# Overexpression of Lipocalin-2 Inhibits Proliferation and Invasiveness of Human Glioblastoma Multiforme Cells by Activating ERK Targeting Cathepsin D Expression

**DOI:** 10.3390/biology10050390

**Published:** 2021-05-01

**Authors:** Yi-Hsien Hsieh, Jen-Pi Tsai, Chen-Lin Yu, Chu-Che Lee, Jen-Chieh Hsu, Jin-Cherng Chen

**Affiliations:** 1 Institute of Medicine, Chung Shan Medical University, Taichung 40201, Taiwan; hyhsien@csmu.edu.tw (Y.-H.H.); s0303019@gm.csmu.edu.tw (C.-L.Y.); koala791029@gmail.com (J.-C.H.); 2 Department of Medical Research, Chung Shan Medical University Hospital, Taichung 40201, Taiwan; 3 Department of Medicine Research, Buddhist Dalin Tzu Chi Hospital, Chiayi 62247, Taiwan; tsaininimd1491@gmail.com (J.-P.T.); dm731849@tzuchi.com.tw (C.-C.L.); 4 Division of Nephrology, Department of Internal Medicine, Dalin Tzu Chi Hospital, Buddhist Tzu Chi Medical Foundation, Chiayi 62247, Taiwan; 5 School of Medicine, Tzu Chi University, Hualien 97071, Taiwan; 6 Department of Neurosurgery, Dalin Tzu Chi Hospital, Buddhist Tzu Chi Medical Foundation, Chiayi 62247, Taiwan

**Keywords:** Lipocalin 2, Cathepsin D, ERK, Glioblastoma multiforme cells

## Abstract

**Simple Summary:**

Lipocalin-2 (LCN2) exhibits pro- and anti-carcinogenic effects in several cancers, but its role in the progression of glioblastoma multiforme (GBM) remains poorly understood. We observed that the overexpression of LCN2 inhibits GBM cell proliferation and invasion via activation of ERK-induced CTSD expression. LCN2 overexpression may be a treatment strategy and prognostic marker for GBM.

**Abstract:**

Lipocalin-2 (LCN2) exhibits pro- and anti-carcinogenic effects in several cancers, but its role in the progression of glioblastoma multiforme (GBM) remains unclear. This study aims to elucidate the effect of LCN2 in human GBM cell, and the mechanism underlying its effects on GBM malignant progression. We observed that LCN2 expression was significantly lower in GBM than in normal tissues and was associated with poorer GBM patient survival. LCN2-overexpressing GBM cells showed significantly reduced proliferation and migration/invasion abilities. Human protease antibody array analysis showed that the expression of cathepsin D (CTSD) protein and mRNA was lower in LCN2-overexpressing GBM cells than in controls. Higher CTSD expression was observed in GBM tumors than in normal tissues, and higher CTSD expression was associated with poorer overall and disease-free survival. LCN2-overexpressing GBM cells exhibited increased ERK phosphorylation. Treatment of these cells with a MEK inhibitor (U0126) restored CTSD expression, cell migration, and cell invasiveness. In conclusion, LCN2 might be serving as a prognostic marker and promising anti-proliferative and anti-metastatic target for treating GBM.

## 1. Introduction

Glioblastoma multiforme (GBM) is the most malignant and frequently occurring type of primary brain tumor [1]. Despite a variety of therapies against GBM including surgery with adjuvant chemo- or radiotherapy, the prognosis is still poor and survival is limited [2]. Recently-developed targeted therapies that are developed to overcome GBM resistance to chemo- or radio-therapy show promising results [3]. A better understanding of the mechanisms underlying GBM progression could promote the development of novel therapeutic choices for better patient outcomes.

Lipocalin-2 (LCN2), also known as neutrophil-gelatinase–associated lipocalin, is a 25 kDa stress protein belonging to the lipocalin superfamily. LCN2 is released under a variety of inflammatory conditions, including obesity-related inflammation in adipose tissue [4]. LCN2 also plays critical roles in the promotion of tumorigenesis by increasing cell proliferation and the metastatic potential of several human tumors [5,6,7,8,9]. Previous studies have shown a positive correlation between the invasive capacity of tumor cells and their expression of LCN2, which promotes the epithelial–mesenchymal transition (EMT) and increases the motility and invasiveness of prostate cancer cells [5]. Microarray analysis showed that LCN2 was the most highly upregulated protein in neoplastic endometrial epithelia, correlating with increased proliferation and invasiveness of endometrial carcinoma cells [7]. In addition, LCN2 over-expression has been observed in carcinoma of the esophagus, breast, and ovary [4,9,10,11,12], and is an independent predictor of overall survival of breast and ovarian cancer [8,12]. In contrast to its pro-oncogenic functions, LCN2 also can exhibit anti-tumorigenic activity. In pancreatic cancer, LCN2 expression suppresses cell adhesion, invasion, and angiogenesis by negatively modulating vascular endothelial growth factor [13]. LCN2 was also observed to decrease the invasiveness and metastasis of malignant colon cells [14]. Furthermore, the under-expression of LCN2 is reported to correlate inversely with the severity of ovarian cancer and progression of the EMT as evidenced by decreased E-cadherin and increased vimentin expression [15]. An in vitro study of GBM showed that the downregulation of LCN2 expression results in chemotherapy resistance [6].

Cysteine cathepsin protease, a lysosomal degradative enzyme, is frequently dysregulated in malignant transformation. This protease promotes tumor progression through increased cell proliferation, invasion, and metastasis and degradation of the extracellular matrix (ECM) in numerous cancers via differing mechanisms [16,17,18]. Increased expression of cathepsin D (CTSD) is reported to correlate with a poor prognosis in esophageal squamous cell carcinoma and malignant glioma [17,18,19]. The importance of CTSD in malignant glioma invasion was demonstrated using mass spectrometry analysis [20] and under- or over-expression of CTSD modulated the malignant potential and radio-sensitivity of glioma cells [21,22,23].

While LCN2 appears to modulate the process of malignant transformation, the detailed mechanism underlying LCN2 action in GBM remains unknown. This study investigates the effect of LCN2 in human GBM cells, its effects on tumorigenesis, and the mechanism underlying its effects on GBM malignant progression.

## 2. Results

### 2.1. Analysis of LCN2 Expression Levels Using Data in the GBM Database and In Vitro Experiments on Malignant Glioma Cell Lines

**Figure 1:**
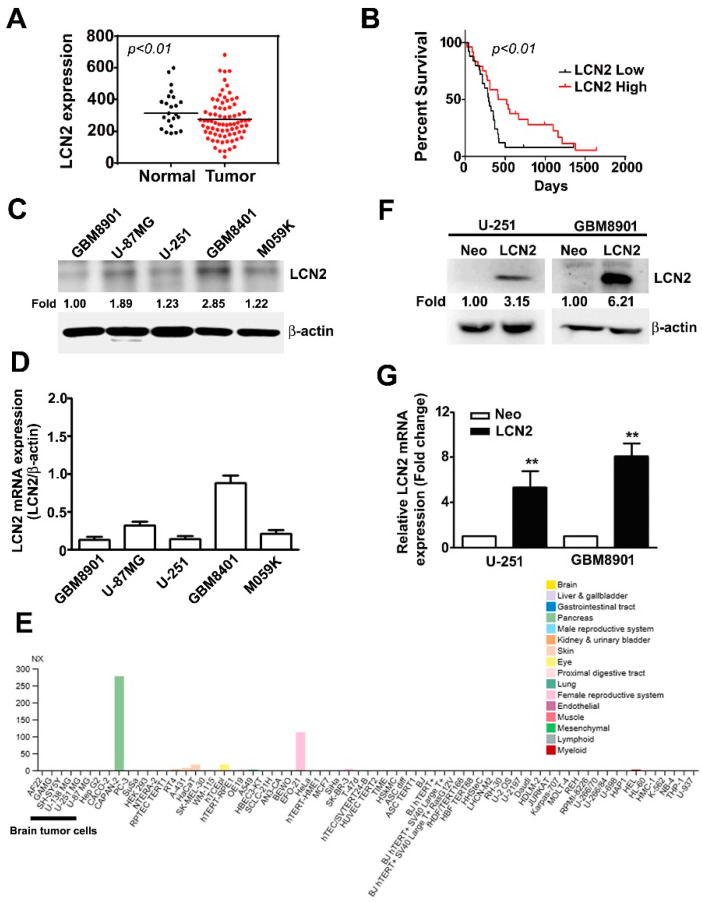
LCN2 expression in GBM tissue, glioma cell lines, and LCN2-transfected GBM cells (**A**) LCN2 expression in GBM and normal tissues and (**B**) survival analysis of data in the GSE4290 and GSE13041 database. Endogenous LCN2 expression in human GBM cell lines (GBM8901, U-87MG, U-251, GBM8401 and M059K) was analyzed by western blot (**C**) and RT-qPCR (**D**). (**E**) The LCN2 mRNA expression level in human GBM cells (GAMG, SH-SY5Y, U-138 MG and U-87MG) from the Human Protein Atlas project database. LCN2 protein and mRNA levels in LCN2-overexpressed or Neo-GBM8901 and U-251 cells as detected by western blot (**F**) and RT-qPCR (**G**). β-actin, protein loading internal control; GAPDH, mRNA control. Data are presented as the mean ± SE of at least three independent experiments. ** *p* < 0.01 compared to Neo cells.

In the GEO database, we observed significantly lower LCN2 expression in GBM tumors than in normal tissues (*p* < 0.01) (Figure 1A). Kaplan–Meier survival analysis and log-rank tests of GBM in the GEO database revealed that GBM with lower LCN2 expression was associated with significantly poorer survival than that with higher LCN2 expression (*p* < 0.01) (Figure 1B). Taken together, these findings suggest that the LCN2 expression level might play an important role in the progression of GBM and serve as an independent prognostic factor for GBM patients. Western blot and RT-qPCR analysis of human malignant glioma cell lines (GBM8901, U-87MG, U-251, GBM8401 and M059K) showed that GBM8901, U-87MG, U-251 and M059K cell lines had lower protein and mRNA expression of LCN2 than the GBM8401 cells (Figure 1C,D). As shown in Figure 1E, using The Human Protein Atlas project database were revealed that low or undetected expression of LCN2 in human GBM cells (GAMG, SH-SY5Y, U-138 MG and U-87MG). Given that we hypothesized that LCN2 plays a role in GBM tumorigenesis, we established LCN2-overexpressing U-251 and GBM8901 cells for further analysis. The results of western blot and RT-qPCR analysis demonstrated upregulated protein and mRNA expression of LCN2 in both cell lines (Figure 1F,G).

### 2.2. Effect of LCN2 Overexpression on Viability and Proliferation of Human Malignant Glioma Cells

**Figure 2:**
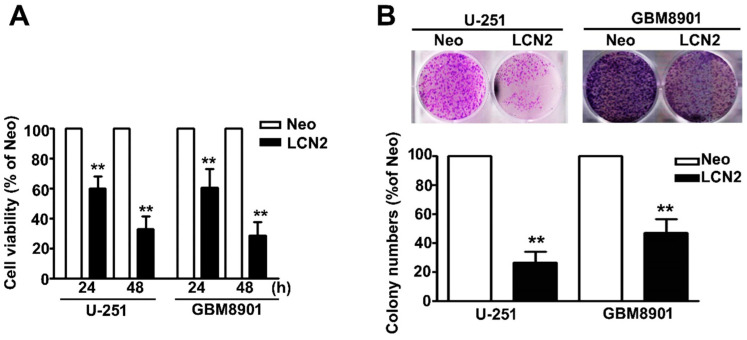
Effects of LCN2 on the cell viability and proliferation of GBM cells (**A**) Viability of LCN2-or Neo-expressing GBM8901 and U-251 cells as determined by MTT assay and (**B**) colony-forming assay. The values are expressed as the mean ± SE of at least three independent experiments. ** *p* < 0.01 compared with that of Neo cells.

The MTT assay was used to further explore the influence of MTA2 on the viability and proliferation of human GBM8901 and U-251 cells. We found that the LCN2-overexpressing GBM8901 and U-251 cells were significantly less viable (Figure 2A). Inhibited proliferation also was observed in LCN2-overexpressing GBM8901 and U-251 cells as compared to Neo-GBM8901 and U-251 cells (Figure 2B). These results suggest that LCN2 may modulate the progression of GBM tumorigenesis.

### 2.3. LCN2 Inhibited the Migration and Invasion of Human Malignant Glioma Cells

**Figure 3:**
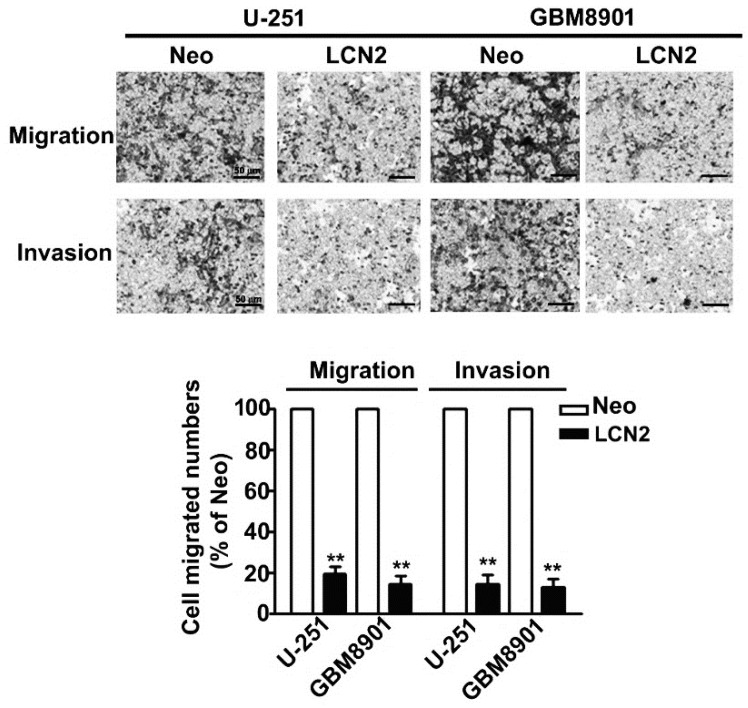
Migration and invasion of LCN2-overexpressing GBM cells. In vitro migration assay and Matrigel-invasion assay were used to assess the migration and invasion ability of LCN2-overexpressing cells. Cells in the lower surface of the Borden chamber were stained and photographed under a light microscope. The number of cells that migrated or invaded were counted, and the results are shown in the histogram in the lower panel. Data are presented as the mean ± SE of at least three independent experiments. ** *p* < 0.01 compared with Neo-expressing cells. Scale bar = 50 μm.

In vitro migration and invasion assays showed that LCN2 overexpression signifi-cantly decreased the migration and invasion of LCN2-overexpressing U-251 and GBM8901 cells compared to that of Neo-U-251 and GBM8901 cells (Figure 3). These results suggest that LCN2 is involved in GBM metastasis.

### 2.4. LCN2 Inhibited the Expression of CTSD of Human GBM Cells and the Clinical Significance of CTSD in GBM Patients

**Figure 4:**
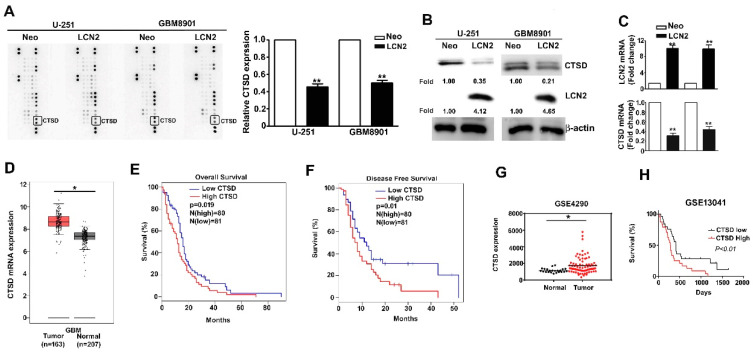
CTSD expression is inhibited by LCN2 overexpression and correlates inversely with GBM patient survival. (**A**) A human protease array profile was performed on whole cell lysate from LCN2-overexpressed or Neo-GBM8901 and U-251 cells. (**B**) The expression of protein and (**C**) mRNA of LCN2-overexpressed or Neo-GBM8901 and U-251 cells were analyzed through western blot and RT-qPCR, respectively. (**D**,**G**) Comparison of CTSD expression level between GBM and normal tissues of GBM patients from the GEPIA and GSE4290 database. β-actin as protein loading internal control. GAPDH as mRNA control. Comparison of the overall survival (**E**,**H**) and disease-free survival (**F**) between low and high expression of CTSD from the GEPIA and GSE13041 database. Data are presented as the mean ± SE of at least three independent experiments. * *p* < 0.05, ** *p* < 0.01 compared with the Neo cells or normal tissues.

CTSD expression in LCN2-overexpressing GBM8901 and U-251 cells was analyzed using a human protease array assay (Figure 4A, left) and qualification of CTSD protein expression (Figure 4A, right). List of quantified proteins expression of human protease protein expression in GBM cells (Supplementary Figure S1). The CTSD protein and mRNA expression was significantly lower in LCN2-expressing cells than in the Neo-expressing counterparts as shown by Western blot (Figure 4B) and RT-qPCR (Figure 4C) analysis. Analysis of data in the GEPIA and GEO database (GSE4290) revealed higher expression of CTSD in GBM tumors than in normal tissues (Figure 4D,G). Kaplan–Meier survival analysis and log-rank tests revealed higher overall and disease-free survival of GBM patients with low CTSD expression than with high CTSD expression (Figure 4E,F, respectively). Similar results were obtained using data in the GEO database (GSE13041) (Figure 4H). Together, these findings suggest that the inhibitory effects of LCN2 on GBM cells are mediated by the suppression of CTSD expression.

### 2.5. Targeting the ERK/CTSD Pathways Involved in LCN2 Inhibit Cell Migration and Invasion of Human GBM Cells

**Figure 5:**
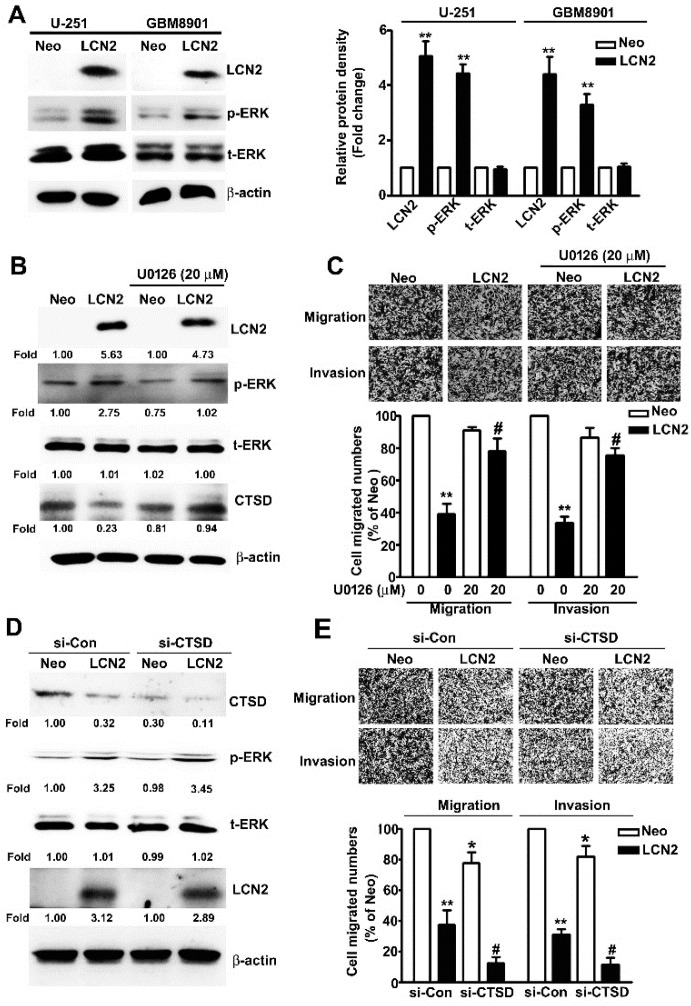
Effect of LCN2 overexpression on ERK-targeted CTSD expression. (**A**) Western blot analysis of total lysates of LCN2-overexpressing or Neo-GBM8901 and U-251 cells to determine the protein expression of LCN2, p-ERK, and t-ERK (left panel). (**B**) Western blot analysis of the same cells were treated with the MEK inhibitor U0126 (20 μM) for 2 h to determine the protein expression of LCN2, p-ERK, t-ERK, and CTSD (right panel). (**C**) The migration and invasiveness of cells following treatment with or without U0126 (20 µM) in LCN2-overexpressing or Neo-U-251 cells. (**D**) Transfection with si-Con or si-CTSD in LCN2-overexpressing or Neo-U-251 cells for 48 h, then extracted the total proteins and measured by western blot. (**E**) The migration and invasiveness of cells following transfected with si-Con or si-CTSD in LCN2-overexpressing or Neo-U-251 cells. β-actin was used as an internal control for protein loading Data are presented as the mean ± SE of at least three independent experiments. * *p* < 0.05, ** *p* < 0.01 compared with Neo cells or si-Con in Neo overexpressing cells. # *p* < 0.05, compared with LCN2-overexpressing cells or si-Con in LCN2 overexpressing cells.

To determine which ERK target CTSD signaling pathway is involved in the LCN2-induced effects on GBM8901 and U-251 cells, we investigated protein expression and phosphorylation in these cell lines. The LCN2-overexpressing cells exhibited significantly higher levels of phosphorylated ERK (Figure 5A) in Western blot analysis. The treatment of U-251 cells with the MEK inhibitor U0126 resulted in significant down-regulation of ERK phosphorylation, restored the expression of CTSD (Figure 5B), and restored their migration and invasive abilities (Figure 5C). To clarify the role of ERK/CTSD expression is LCN2-overexpressing cells, using the knockdown assay to found that treatment of siRNA-CTSD (si-CTSD) in LCN2-overexpressing U-251 cells resulted in significant inhibition of CTSD expression, not affect the phosphorylated ERK expression (Figure 5D), and inhibited the cell migration and invasive abilities (Figure 5E), compared with si-Con in LCN2-overexpressing U-251 cells. This result suggests that CTSD is key regulator in LCN2 inhibit cell migration and invasion of GBM cells. These results clearly indicate that LCN2 inhibited the CTSD expression by upregulating the ERK signaling pathway, which plays a key role in inhibiting the migration and invasiveness of human GBM cells.

## 3. Discussion

In this study, we found that (1) GBM patients with lower LCN2 expression and higher CTSD expression had a poorer prognosis; (2) LCN2 expression inhibited the cytotoxic effects in GBM cells; (3) overexpression of LCN2 inhibited the invasion and migration of malignant GBM cells in vitro; (4) overexpression of LCN2 exerted anti-metastatic effects by suppressing CTSD expression via upregulation of the ERK signaling pathway. Despite international efforts to develop GBM treatments, GBM survival remains low [24]. GBM management has evolved from surgery followed by adjuvant chemotherapy or radical radiotherapy to targeted therapies, but the prognosis is still disappointing [25]. Considering the associated side effects of modern chemotherapeutic agents and with the high rate of GBM progression or recurrence after treatment, the identification of new therapeutic targets for GBM is needed.

LCN2 is known to play dual roles in mediating the progression of tumorigenesis. One role involves the promotion of tumorigenesis by increasing cell proliferation, the metastatic potential and independent predictor for severity of grades and stages of tumors [26,27]. In vitro and in vivo studies have shown that LCN2 promotes cell invasion and migration and distant metastasis in prostate cancer via the ERK signaling pathway, causing up-regulation of the SLUG transcription factor to induce the EMT [5]. An LCM-microarray study showed that the LCN2 expression was upregulated during the malignant process of endometrial carcinoma, and the forced expression of LCN2 promoted cell growth and increased the invasive potential of endometrial carcinoma cells. In esophageal squamous cell carcinoma, LCN2 expression is modulated by the transcription factors TCF7L2 and EGR1, leading to increased activity of matrix metalloproteinase-9 (MMP-9), rearrangement of cytoskeletal F-actin, as well as increased invasiveness and migration through the MEK/ERK pathway [9,10]. In stark contrast to its pro-oncogenic function, LCN2 is reported to suppress tumorigenesis in pancreatic, colon, and ovarian cancer [13,14,15,28]. Tong et al. observed higher LCN2 expression in well- to moderately-differentiated prostate cancer cells than in those that were poorly differentiated. In addition, the over-expression of LCN2 inhibited cell adhesion, invasion, and angiogenesis by suppressing the activation of focal adhesion kinase and vascular endothelial growth factor [13]. In addition, they found that epidermal growth factor inhibited the expression of E-cadherin and LCN2 through the MEK/ERK signaling pathway [28]. In addition, the under-expression of LCN2 was correlated inversely with the severity of ovarian cancer, and in vitro study showed marked enhanced EMT and cellular spread after epidermal growth factor induced inhibition of LCN2 expression [15]. In oral squamous cell carcinoma, the knockdown of LCN2 correlated with increased cancer cell malignant potential and resistance against chemotherapy with upregulated MMP-9 expression [29]. In an in vitro study of GBM, LCN2 expression correlated negatively with malignancy and resistant to chemotherapy; additionally, the transfection of BCNU-resistant variant glioma cells with LCN2 cDNA resulted in apoptotic sensitivity and chemosensitivity through inhibition of the Akt signaling pathway [6]. In this study, we observed lower LCN2 expression levels in GBM tumors than in normal tissues and found that lower LCN2 expression correlated with poorer survival in GBM patients. In addition, upregulated LCN2 expression led to decreased tumor malignant potential, proliferation, invasiveness, and migration ability through upregulation of the ERK signaling pathway. Together, these findings suggest that whether LCN2 functions in a pro- or anti-tumorigenesis manner is cell-context dependent. LCN2 might act as an inhibitor of the malignant evolution of GBM by mediating the ERK signaling pathway.

Cysteine cathepsin proteases are degradative enzymes that are involved in lysosomal functions such as protein degradation, autophagy, and cell death. These enzymes are also involved in the features of malignant transformation, including altered adhesion, dysregulated degradation of the ECM, invasion into the vasculature, and distant metastasis [30,31]. CTSD is an aspartic endo-protease that ubiquitously distributed in lysosomes. CTSD overexpression is reported to be associated with higher recurrence rates and shorter disease-free and overall survival in various tumor cells [32]. Mass spectrometry analysis showed that CTSD was important in the invasiveness of malignant glioma cells [20] and increased CTSD expression correlates with more advanced GBM grade and poorer survival [18,19]. These findings suggest that CTSD might be a useful GBM marker and predictor of prognosis. Moreover, in vitro studies have shown that the manipulated inhibition or promotion of CTSD expression modulates apoptosis, proliferation, invasiveness, migration ability, and autophagy level to affect the radio-sensitivity of glioma cells [21,22,23]. Our results found that migration and invasion significantly inhibition of si-Control-LCN2 or si-CTSD-Neo treatment upon LCN2 overexpressing GBM cells, but not completely inhibition of CTSD protein expression (Figure 5D). Based on these observations, coupled with the relationship between LCN2/CTSD on tumor invasion progression, we speculated that the anti-invasive effect of LCN2 on GBM cell may be partly through CTSD expression and possibly involved in another molecular mechanism. Therefore, other important key molecules for LCN2 regulate GBM cell migration and invasion, which will be a focus in detailing the molecular mechanism and requires further investigation.

In this study, we found that LCN2 over-expression in GBM cells decreased CTSD expression by mediating the level of ERK phosphorylation, leading to a decrease in their malignant potential. Together, our findings indicate that p-MEK/p-ERK-mediated expression of CTSD could play an important role in malignant transformation and the invasive potential of GBM cells. Although debate continues regarding the pro- or anti-neoplastic role of LCN2 in a variety of cancers, our findings suggested that LCN2 may be a promising anti-proliferative and anti-metastatic target for treating GBM in the future.

## 4. Conclusions

Taken together, these findings show that LCN2 clearly plays a key role in the physiopathology of malignant processes in GBM. To the best of our knowledge, this study is the first to show the anti-proliferative and anti-metastatic effects of LCN2 targeting ERK/CTSD pathways on human GBM cells in vitro.

## 5. Methods and Materials

### 5.1. Cell Culture and Reagents

The human glioma U-251 cell line was kindly provided by Dr. Dah-Yu Lu (Department of Pharmacology, School of Medicine, China Medical University, Taichung, Taiwan). The human glioma GBM8401 and GBM8901 cell line was kindly provided by Dr. Li-Sung Hsu (Institute of Medicine, Chung Shan Medical University, Taichung, Taiwan). The U-87MG (BCRC No. 60360) and M059K cell line (BCRC No. 60381) were purchased from the Bioresources Collection and Research Center (BCRC, Hsinchu, Taiwan). These cells were cultured in DMEM/F12 and MEM medium supplemented with 1 × penicillin/streptomycin and 10% fetal bovine serum (HyClone, Logan, UT, USA) were incubated at 37 °C in a humidified incubator containing a 5% CO_2_ atmosphere. LCN2 antibody was purchased from R&D Systems, Inc (Minneapolis, MN, USA). U0126 (MEK inhibitor), siRNA-CTSD (si-CTSD), Cathepsin D (CTSD), t-ERK, β-actin, HRP-mouse and HRP-rabbit antibodies were purchased from Santa Cruz Biotechnology (Santa Cruz, CA, USA). Matrigel Matrix was purchased from Corning company (Tewksbury, MA, USA). Phosphorylated-ERK (p-ERK) was obtained from Cell Signaling Technology (Beverly, MA, USA).

### 5.2. Assessment of Cell Growth

Cell growth was measured using the MTT reagent (Millipore-Sigma, St. Louis, MO, USA) as followed previously report [33]. Neo- and LCN2-GBM cells were incubated for 24 or 48 h, followed by addition of the MTT reagent (0.5 mg/mL) in RPMI or MEM me-dium and incubation for 4 h. The medium was removed, and cells were stained in crystal violet with isopropanol at room temperature for 5 min. The absorbance of each sample was recorded at 570 nm using a microplate reader (Bio-Rad, Hercules, CA, USA) to calculate the cell growth. All cell growth assays were triplicate.

### 5.3. Cell Proliferation Assay

Briefly, Neo- and LCN2-GBM cells were seeded into 6-well culture dishes (1 × 10^4^ cells/well) and incubated in culture medium for 24 h. The culture medium was replaced every 3 days. After incubating for 10 days, the colonies were fixed with 4% formaldehyde for 15 min, stained with 0.5% crystal violet for 30 min, and then washed twice with PBS. The cells were photographed, and the number of colonies was counted twice. The experiments were performed in triplicate.

### 5.4. In Vitro Migration and Invasion Assay

The migration ability of Neo- and LCN2-GBM cells was determined in vitro migration and invasion assay. Neo- and LCN2-GBM cells (3 × 10^5^ cells/well) were pre-coated with or without Matrigel Matrix on 48-well modified Boyden chambers with 8 μm pore size membranes and incubated for 18 h (migration) or 24 h (invasion). The membrane was then fixed in methanol for 15 min and soaked in 1% crystal violet solution for 30 min. Cell migration was quantified and photographed under a light microscope (Olympus, Tokyo, Japan).

### 5.5. Human Proteinase Assay

The Proteome Profiler Human Protease Array (R & D, Minneapolis, MN, USA) was used to assay total cell lysates (Neo- or LCN2-GBM cells) according to the manufacturer’s instructions. The protease expression profile analysis was conducted as previously re-ported [34].

### 5.6. Western Blotting

The total cellular proteins of Neo- and LCN2-GBM cells were extracted using lysis buffer and quantified using the Bradford assay (BioRad, Hercules, CA, USA). Total cell lysates were separated by SDS-PAGE and transferred to 0.22 µm Immobilon polyvinylidene fluoride membranes (Millipore, Burlington, MA, USA). The membranes were incubated in blocking buffer (5% skim milk with TBST buffer) for 30 min, followed by the addition of antibodies and incubation overnight. After incubation with secondary HRP-mouse or HRP-rabbit antibodies (Santa Cruz Biotechnology, Santa Cruz, CA, USA), immunoblot bands were visualized using Immobilon HRP Substrate (Millipore, Darmstadt, Germany), photographed, and the image analyzed using an ImageQuant LAS-4000 mini Analyzer (GE Healthcare, Marlborough, MA, USA).

### 5.7. siRNA Transfection Assay

The siRNA-control (si-Con) or siRNA-CTSD (si-CTSD) were obtained from Santa Cruz Biotechnology (CA, USA). The Neo- and LCN2-U-251 cells were transfected with the si-Con or si-CTSD by using a TurboFect™ Transfection Reagent (Thermo Fisher Scientific, MA USA) following manufacturer’s instruction. After 48 h, the total lysate was extracted and analyzed with western blotting, and detected cell migrate numbers by migration and invasion assay.

### 5.8. Clinical Data from TCGA Database

Two datasets of interest in the LCN2 and CTSD GEO platform were selected (GSE4290, GSE13041) and analyzed for clinical tissue expression and overall survival by using Kaplan-Meier Plotter and the GEPIA database. The LCN2 mRNA expression level in human GBM cells was analyzed by The Human Protein Atlas project database.

### 5.9. Statistical Analysis

Data are presented as the mean ± standard error of three independent experiments. Analysis of variance (ANOVA) and Student’s *t*-test were used to determine the significance of differences. *p* < 0.05 or *p* < 0.01 was considered significant.

## Data Availability

The authors will freely release all data underlying the published paper upon direct request to the corresponding author.

## Supplementary Materials

The following are available online at https://www.mdpi.com/article/10.3390/biology10050390/s1, Figure S1: The list of quantified these protein expressions in Neo-overexpressed or LCN2-GBM8901 and U-251 cells by human protease array profile.
