# Supplementary material for: Overexpression of Lipocalin-2 Inhibits Proliferation and Invasiveness of Human Glioblastoma Multiforme Cells by Activating ERK Targeting Cathepsin D Expression
Source: Biology (Basel). 2021 May 1;10(5):390. doi: 10.3390/biology10050390 (PMC8147321; doi:10.3390/biology10050390)
Supplement: Supplementary file 1 [file biology-10-00390-s001.zip › biology-1159213-final supplementary/Supplementary Figure S1.pdf]

Supplementary Figure 1

		U-251		GBM8901	
		**Neo**	**LCN2**	**Neo**	**LCN2**
A5, A6	ADAM9 8754 Ectodomain MDC9, meltrin g	1.00	0.99	1.00	1.12
A7, A8	ADAMTS1 9510 Proform METH1	1.00	0.95	1.00	1.11
A9, A10	ADAMTS13 11093 Active von Willebrand factor-cleaving protease	1.00	0.64	1.00	0.90
A11, A12	Cathepsin A 5476 Proform & Active CTSA, Lysosomal Carboxypeptidase A	1.00	0.94	1.00	0.94
A13, A14	Cathepsin B 1508 Proform CTSB, APPS, CPSB	1.00	1.45	1.00	0.63
A15, A16	Cathepsin C 1075 Proform & Active CTSC	1.00	1.43	1.00	0.95
A17, A18	Cathepsin D 1509 Proform & Active CTSD, CPSD	1.00	0.59	1.00	0.51
A19, A20	Reference Spots N/A N/A RS	1.00	1.02	1.00	1.00
B5, B6	Cathepsin L 1514 Proform & Active CTSL, CATL, MEP, Cathepsin L1	1.00	0.98	1.00	1.42
B7, B8	Cathepsin S 1520 Proform & Active CTSS	1.00	1.22	1.00	0.90
B9, B10	Cathepsin V 1515 Proform & Active CTSV, CTSL2, CTSU, Cathepsin L2	1.00	1.12	1.00	0.91
B11, B12	Cathepsin X/Z/P 1522 Proform & Active CTSX, CTSZ	1.00	1.21	1.00	1.06
B13, B14	DPPIV/CD26 1803 Ectodomain ADABP, ADCP2	1.00	1.24	1.00	1.03
B17, B18	Kallikrein 5 25818 Proform & Active KLK5, SCTE, KLKL2	1.00	1.41	1.00	1.22
C7, C8	Kallikrein 10 5655 Proform & Active KLK10, NES1, PRSSL1	1.00	0.67	1.00	1.62
C11, C12	Kallikrein 13 26085 Proform & Active KLK13, KLKL4	1.00	1.30	1.00	0.93
C13, C14	MMP-1 4312 Proform & Active Collagenase 1, Interstitial Collagenase	1.00	1.88	1.00	0.97
C15, C16	MMP-2 4313 Proform & Active Gelatinase A	1.00	1.01	1.00	0.96
C17, C18	MMP-3 4314 Proform & Active Stromelysin-1	1.00	1.45	1.00	0.95
D3, D4	MMP-7 4316 Proform & Active Matrilysin, PUMP 1	1.00	1.09	1.00	1.33
D5, D6	MMP-8 4317 Proform & Active Collagenase 2, Neutrophil Collagenase	1.00	1.21	1.00	1.29
D7, D8	MMP-9 4318 Proform & Active Gelatinase B, CLG4B, GELB	1.00	1.04	1.00	1.55
D9, D10	MMP-10 4319 Proform & Active Stromelysin-2	1.00	0.87	1.00	1.34
D11, D12	MMP-12 4321 Proform & Active Macrophage Elastase	1.00	1.56	1.00	0.82
D13, D14	MMP-13 4322 Proform Collagenase 3	1.00	0.96	1.00	0.79
D15, D16	Neprilysin/CD10 4311 Ectodomain MME, NEP, CALLA	1.00	1.54	1.00	0.87
D17, D18	Presenilin 5663 N-Terminal Fragment PSEN1, AD3, PS-1	1.00	1.29	1.00	0.86
E1, E2	Reference Spots N/A N/A RS	1.00	1.09	1.00	1.05
E7, E8	uPA/Urokinase 5328 Proform & Active Urokinase-type Plasminogen Activator, PLA	1.00	1.06	1.00	1.21

Supplementary Figure S1: The list of quantified these protein expressions in Neo-overexpressed or LCN2-GBM8901 and U-251 cells by human protease array profile.

.
