# Supplementary figures and images for: Overexpression of Lipocalin-2 Inhibits Proliferation and Invasiveness of Human Glioblastoma Multiforme Cells by Activating ERK Targeting Cathepsin D Expression
Source: Biology (Basel). 2021 May 1;10(5):390. doi: 10.3390/biology10050390 (PMC8147321; doi:10.3390/biology10050390)

**Figure-1C**

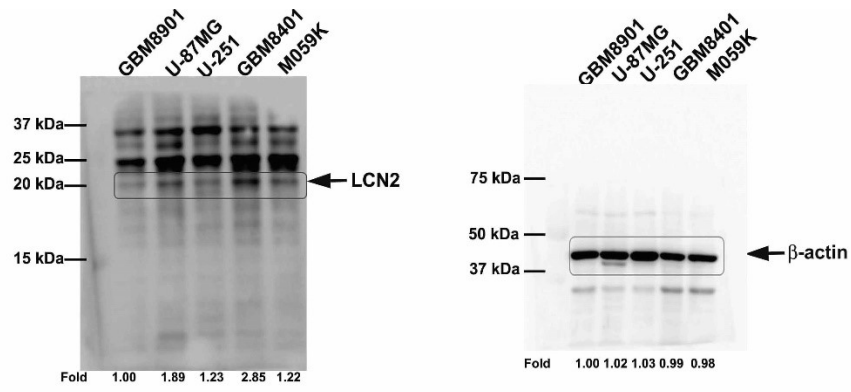

**Figure-1E**

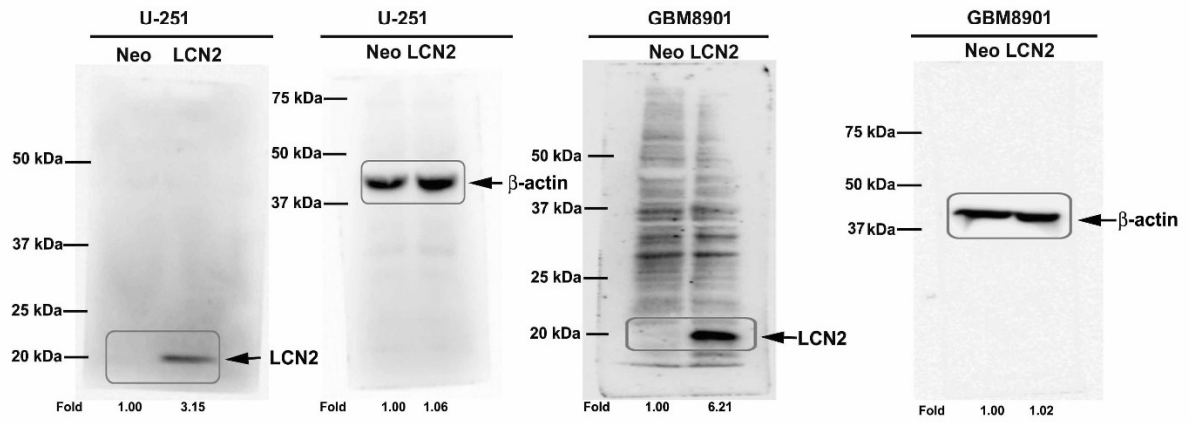

Figure-4B

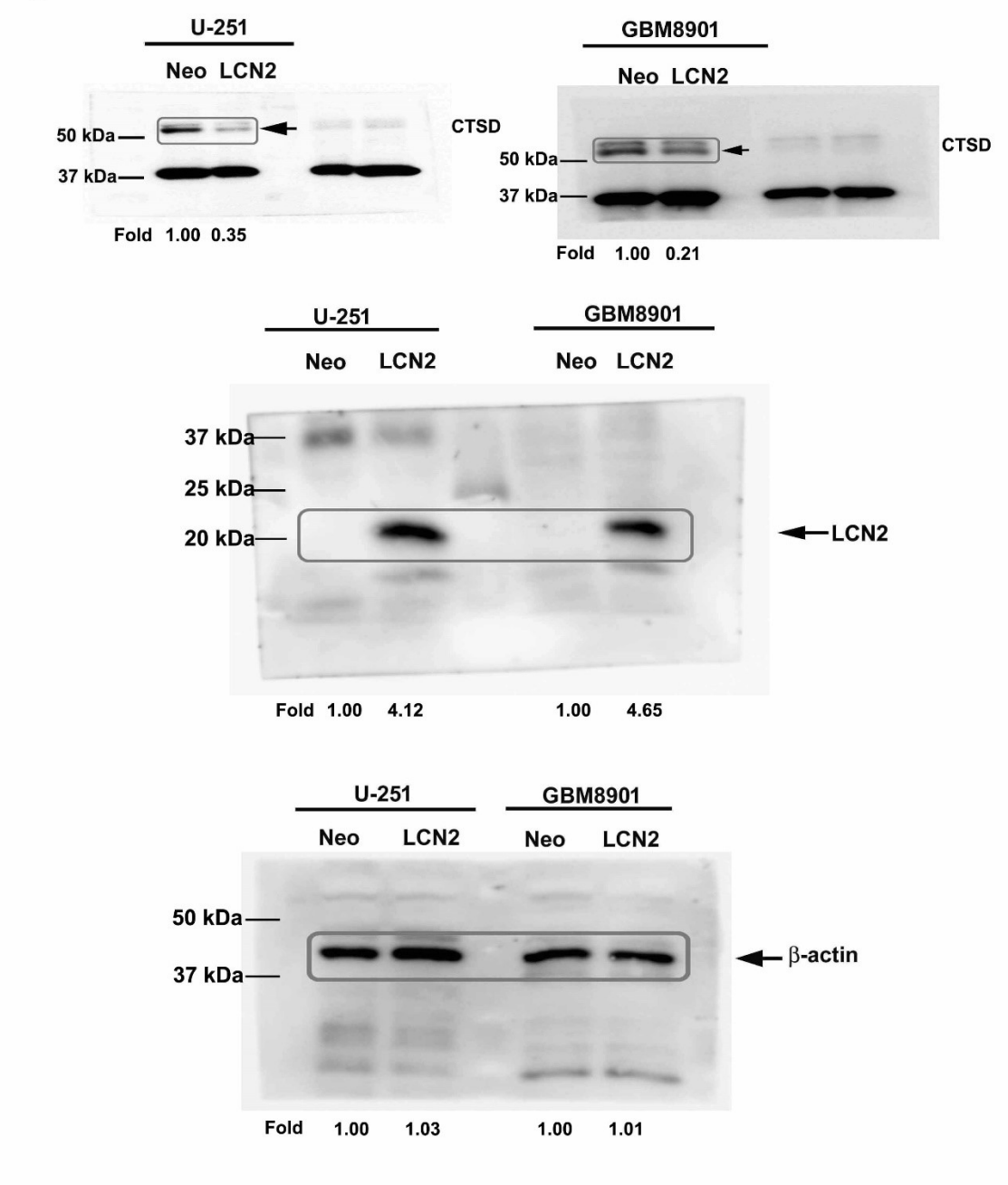

Figure-5A

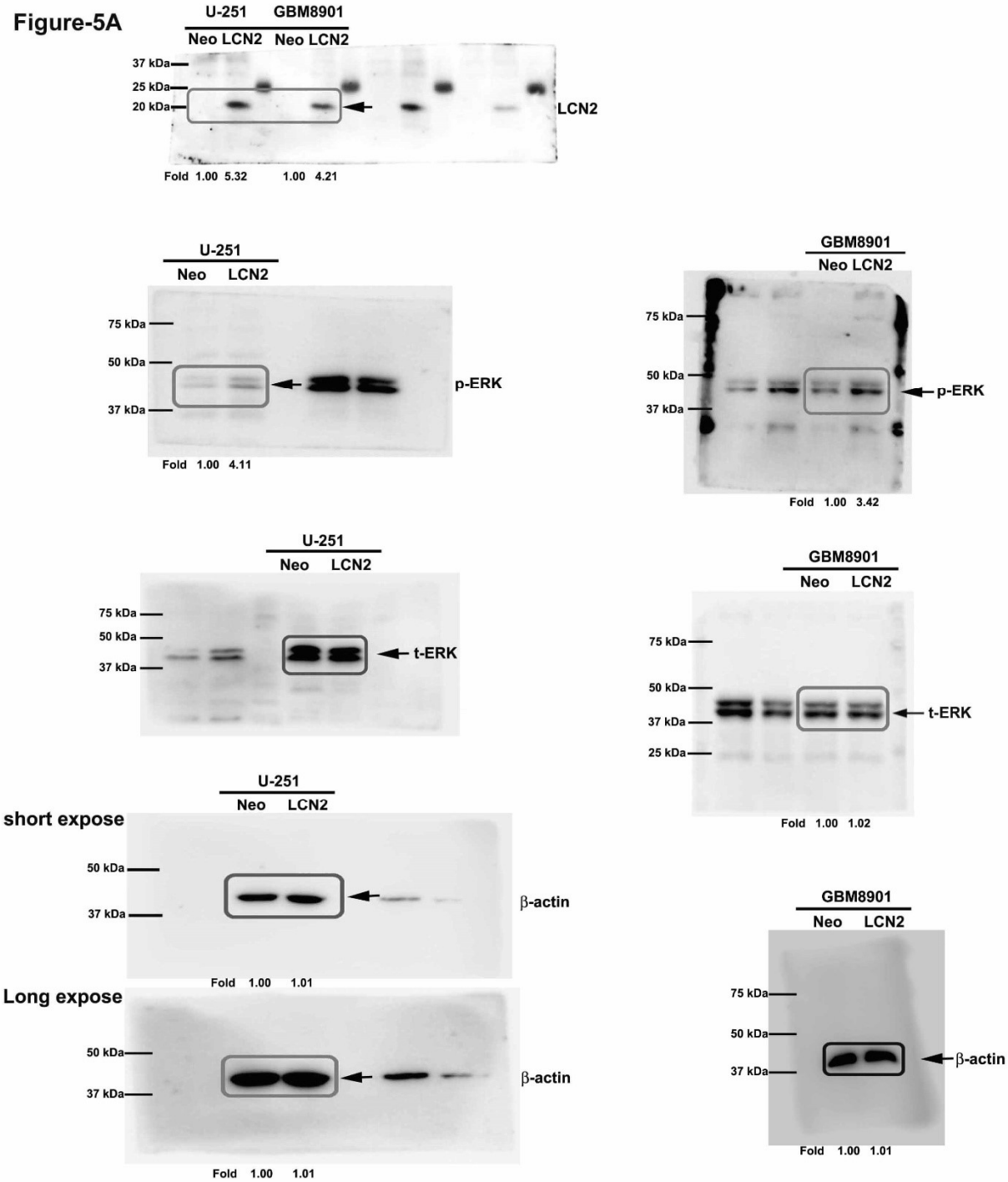

Figure-5B

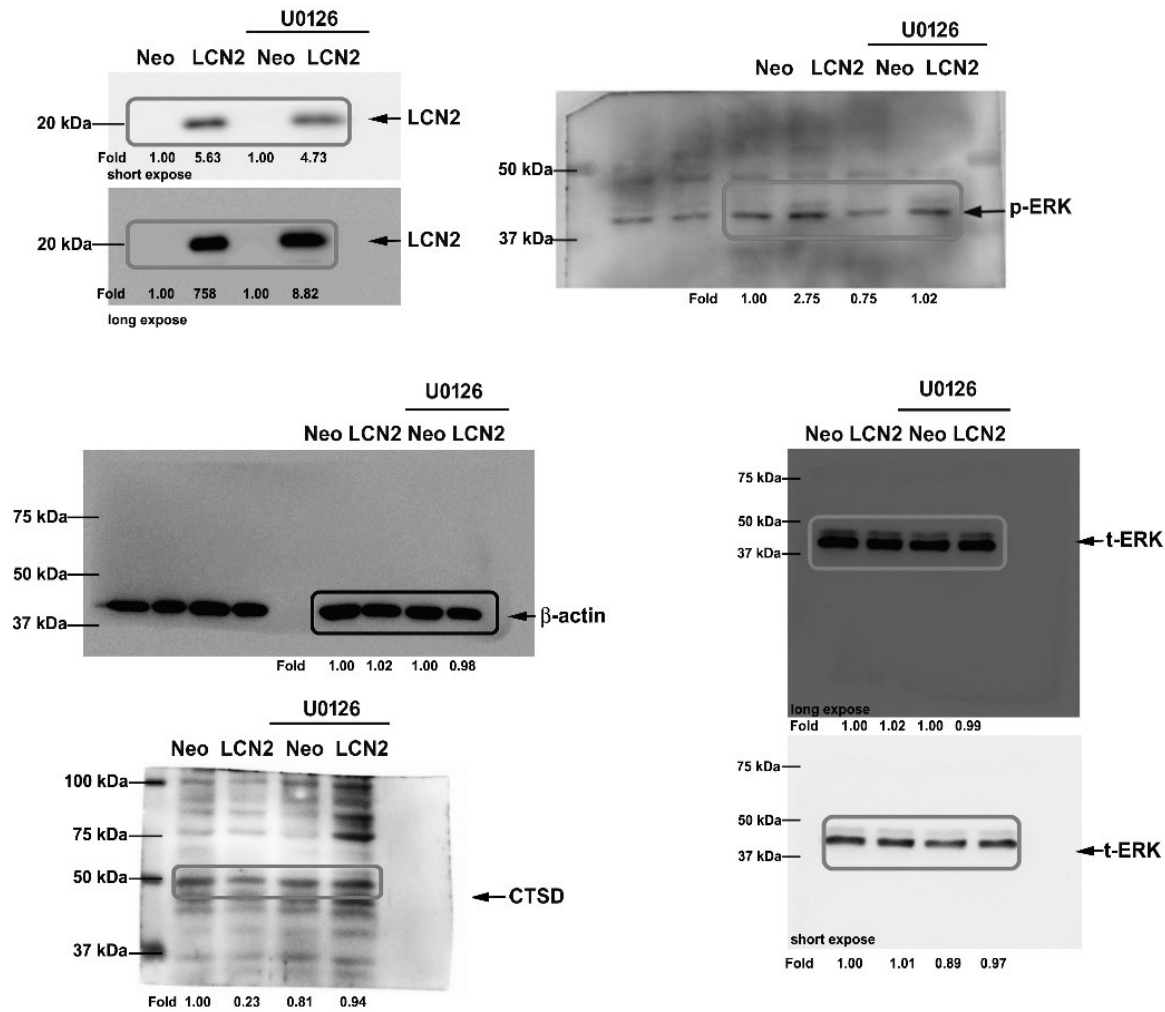

**Figure-5D**

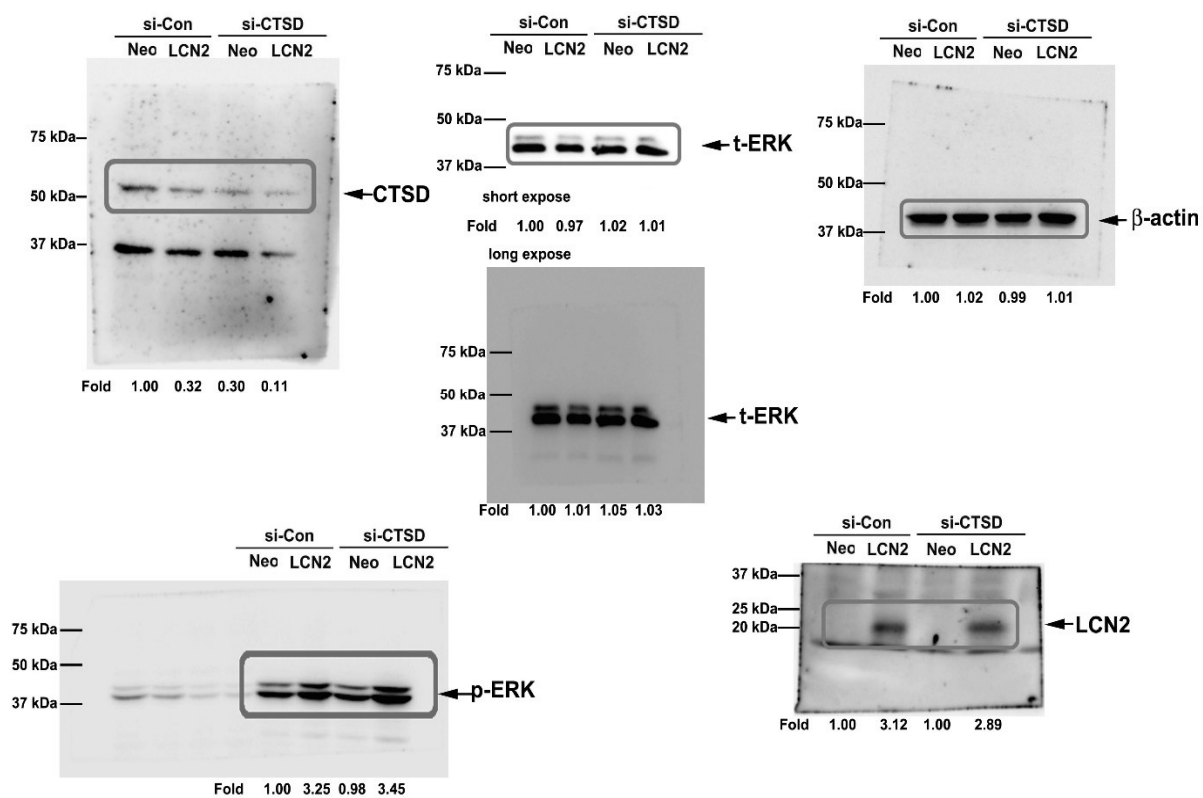

Supplement: Supplementary file 1 [file biology-10-00390-s001.zip › biology-1159213-final supplementary/origin data.pdf]
